# Impact of web-based health education on HPV vaccination uptake among college girl students in Western and Northern China: a follow-up study

**DOI:** 10.1186/s12905-022-01625-0

**Published:** 2022-02-23

**Authors:** Xi Zhang, Hui Chen, Jing Zhou, Qian Huang, Xiao-yu Feng, Jing Li

**Affiliations:** 1 Key Laboratory of Carcinogenesis and Translational Research (Ministry of Education), Beijing Office for Cancer Prevention and Control, Peking University Cancer Hospital & Institute, Beijing, 100142 China; 2 Women’s Hospital, Zhejiang University School of Medicine, Hangzhou, 310006 China; 3 Chenghua District Center for Disease Control and Prevention, Chengdu, 610057 China; 4 Student Affairs Department, West China School of Public Health and West China Fourth Hospital, Sichuan University, Chengdu, 610041 China; 5 Shanxi University of Finance and Economics, Taiyuan, 030006 China; 6 West China School of Public Health and West China Fourth Hospital, Sichuan University, Chengdu, 610041 China

**Keywords:** Web-based health education, HPV vaccine uptake, Female college students, Knowledge and attitudes, Influencing factors

## Abstract

**Objective:**

To investigate the effect of a web-based educational intervention on changing female college students’ willingness and uptake of human papillomavirus (HPV) vaccines, and factors associated with HPV vaccination acceptance in Western and Northern China.

**Methods:**

A web-based cluster randomized controlled trial was conducted in Western and Northern China from February to May 2020. A total of 967 female freshmen were recruited from two universities through convenience sampling, stratified sampling (liberal arts or sciences), and cluster sampling. Eligible students were randomized into intervention and control group with a 1:1 allocation ratio. The intervention group received seven days of web-based health education regarding HPV and HPV vaccines, whereas the control group received non-HPV-related materials. All students were asked to complete a post-intervention questionnaire to measure their awareness, uptake, and willingness to receive HPV vaccination at 7-day and one-month intervals. The *chi*-square test and Student’s *t-*test were employed to examine the differences between the intervention and control groups for categorical and continuous data. Logistic regressions were used to analyze factors associated with vaccination intentions.

**Results:**

Nine hundred forty-six female freshmen aged 18.99 ± 0.63 years were enrolled in the study, with 532 in the intervention group and 414 in the control group. Prior to the intervention, 63.8%, 66.3%, and 60.8% of students had heard of HPV, HPV-related diseases, and HPV vaccines, respectively. Only 2.2% of students reported being vaccinated, but 33.0% were willing to be vaccinated against HPV. After seven days of education, students in the intervention group exhibited higher awareness (*p* < 0.001) and knowledge scores (5.13 ± 1.23 vs. 3.10 ± 1.99, *p* < 0.001) than those in the control group. Similarly, in the intervention groups, willingness to be vaccinated against HPV was significantly higher than in the control groups (*p* < 0.001). The high cost (57.7%) and concerns about adverse events (56.0%) were the main reasons female college students did not accept HPV vaccines. School location in urban areas, parents’ higher education backgrounds, history of HPV vaccination counseling, history of sexual behavior, and having heard of HPV vaccines were associated with a higher willingness to be vaccinated.

**Conclusion:**

Female college students’ HPV vaccination uptake is insufficient, and they have minimal detailed knowledge about HPV and its vaccines. Web-based health education on HPV vaccines is an easy, feasible, and effective way to improve the awareness and acceptance of HPV vaccination among female college students, but it has limited effect on HPV vaccination uptake.

**Supplementary Information:**

The online version contains supplementary material available at 10.1186/s12905-022-01625-0.

## Introduction

Cervical cancer is the fourth most frequently diagnosed malignant tumor affecting women's health worldwide, with an estimated 604,000 new cases and 342,000 deaths reported in 2020 [1]. China, the most populous country in the world, also bears the most significant global burden of cervical cancer, with 98,900 new cases and 30,500 deaths annually, according to the national cancer registry of China [2].

Prophylactic human papillomavirus (HPV) vaccination can effectively prevent approximately 70–80% of the corresponding HPV infections and reduce the risk of cervical precancer lesions, cervical cancer, and other HPV-related diseases [3, 4]. Since 2016, imported bivalent, quadrivalent, 9-valent, and domestic-made bivalent HPV vaccines have been successively approved by the Chinese authorities for marketing in the Chinese mainland. However, due to the 10-year delay in introducing the HPV vaccine, public awareness and acceptance toward HPV vaccines are generally low, with only approximately 3% vaccination rates among females aged 18–45 years [5].

College students are becoming sexually active and are prioritized as HPV vaccination catch-up groups in developed countries such as the United States [6]. Previous studies have shown that women aged 15–19 are among the peak populations of high-risk HPV infection in China, most of whom are in college [7]. However, Chinese female college students have insufficient knowledge of HPV vaccines, with less than half (44.17%) having heard of HPV vaccines [8], which was significantly lower than that in Western countries (71.4%) [9]. Moreover, the uptake of HPV vaccines was only 11% in female college students [10], far below the World Health Organization (WHO) goal of eliminating cervical cancer by 2030 [11]. Therefore, it is particularly urgent to carry out health education on HPV vaccination among female college students in the mainland of China.

Numerous studies have demonstrated that HPV-related health education can significantly increase awareness of HPV and HPV vaccines, potentially increasing their willingness to receive the HPV vaccine [12–14]. However, most of the target population was general women; few studies have focused on college-aged girls. Furthermore, traditional school-based health education has encountered challenges in implementation due to the COVID-19 pandemic since 2020. Therefore, new health education models for HPV and HPV vaccines among female college students urgently need to be implemented and evaluated.

In the present study, we sought to examine the effect of a web-based educational intervention that affected female college students’ willingness and uptake toward HPV vaccines immediately and one month later, as well as to explore the factors that influence HPV vaccination willingness.

## Methods

### Study design and participants

This study was a web-based, cluster randomized controlled trial involving a 7-day intervention with a further 1-month follow-up conducted in Western (Sichuan Province) and Northern (Shanxi Province) China between February and May 2020. In each province, one comprehensive university was selected through convenience sampling. After that, six colleges from the university in Sichuan Province and two colleges from the university in Shanxi Province were invited using convenience sampling, stratified sampling (liberal arts or sciences), and cluster sampling.

Eligible female freshmen were invited to participate in this study. The inclusion criteria were as follows: (1) females aged 18 years or older; (2) first-year undergraduate students; (3) no vaccination contraindications; and (4) having a mobile phone. The exclusion criteria were as follows: (1) males; (2) females under 18; (3) medical students; and (4) previous history of vaccination contraindications.

### Sample size calculation

We used the formula $N=\frac{{\left[{Z}_{1-\alpha /2}\sqrt{\left({P}_{1}+{P}_{2}\right)\left(1-\frac{{P}_{1}+{P}_{2}}{2}\right)}+{Z}_{\beta }\sqrt{{P}_{1}\left(1-{P}_{1}\right)+{P}_{2}\left(1-{P}_{2}\right)}\right]}^{2}}{{({P}_{1}-{P}_{2})}^{2}}$ to calculate the sample size. PASS software version 14 (NCSS, Kaysville, UT) was performed to estimate that a sample size of 197 participants in the intervention and control groups would be required to achieve 80% power at a 2-sided 5% level of significance to detect a net difference of at least 10% in the uptake rate between the two groups, assuming that the rate is 11% for the control group [10]. After adjustment for a drop-out rate of 20% after enrollment, a total of 237 students per arm, 474 students in each school, and 948 students in total were enrolled.

### Study procedure

Since this study was conducted during the COVID-19 pandemic, all processes were conducted online, including informed consent, baseline survey, intervention, and follow-up survey. Electronic informed consent was obtained from each student before enrollment, followed by an online baseline questionnaire. Furthermore, eligible students were randomly allocated to either the intervention or control group. Random numbers were generated using SAS software version 9.4 (The SAS Institute, Cary, NC) to assign each student individually to either the intervention or control group with a 1:1 allocation ratio with majors as a blocking factor.

**Fig. 1:**
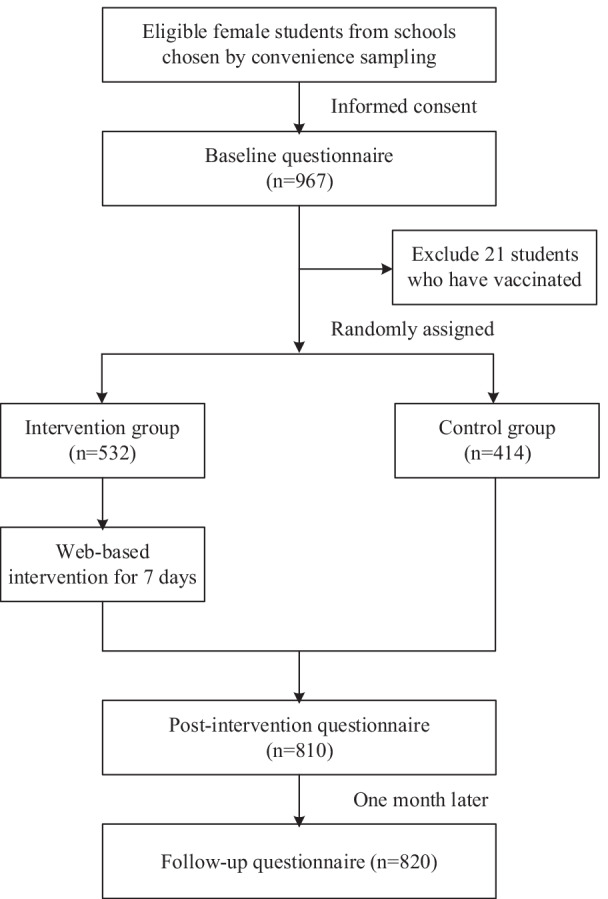
Study flowchart

Participants randomly assigned to the intervention group participated in a 7-day intervention and were asked to complete a post-intervention questionnaire to assess their respective knowledge of HPV and HPV vaccines. Students randomly assigned to the control group were provided with educational materials unrelated to HPV prevention during the same period. They were asked to complete the same questionnaire as the intervention group. Additionally, one month after the intervention, students in both the intervention and control groups received a post-intervention questionnaire on the same day. A mobile application collected all data, and the 7-day intervention was administered through intelligent devices, such as a computer or a mobile phone. The study flowchart is presented in Fig. 1, which contains more detailed information.

### Intervention

The intervention group received a 10-min daily web-oriented multicomponent education for one week, whereas the control group received some health tips about COVID-19, which was not relevant to HPV vaccination. The intervention covered the accompanying themes: (1) general health education on the prevention of vaccination and sexual health; (2) information on HPV infection and its risk factors, cervix and cervical diseases; (3) presentation of HPV immunization as a preventive measure for cervical cancer; (4) case study showing the prognosis of Chinese women with advanced cervical cancer; (5) storytelling the experience of a female undergrad from being ignorant about HPV to settling on a decision to vaccinate against HPV; (6) information on the best way to improve self-efficacy and self-determination to improve healthy behavior; and (7) social strategies for HPV vaccination, including information on the accessibility and price of HPV vaccine at neighborhood facilities.

### Measures

A self-administered electronic questionnaire in Mandarin was used throughout the study to assess socio-demographic information and knowledge and attitudes toward the HPV vaccine. The research team developed this questionnaire after reviewing relevant literature and conducting several rounds of panel discussions. It has already been used and tested in previous studies employed with different populations. In brief, the questionnaire consisted of four subscales: (1) general information about the study participants, including the participants’ HPV vaccination status, socio-demographic characteristics (date of birth, ethnicity, place of residence, parents’ education level, and personal living expenses), and awareness of HPV, HPV-related diseases, and HPV vaccines. (2) perceptions of sexual health education (3 items), including “Have you received sexual health education before the survey?”, “Have you ever had sexual experience?” and “Are you single?” (3) knowledge of HPV and HPV vaccine (7 items) by judging true, false, and don’t know, with a statement including “Persistent HPV infection can lead to cervical cancer.”, “HPV is mainly transmitted through sexual contact.”, “Condoms can reduce HPV infection.”, “HPV infection is almost asymptomatic.”, “HPV infection can cause oral cancer, anal cancer, and genital warts.”, “HPV infection is common.”, “The best time to get vaccinated is before the first debut of sex.” Correct responses for each domain were summed yielding scores of 1 for knowledge of HPV and HPV vaccines. (4) willingness to receive the HPV vaccine (3 items), including “Are you willing to get the HPV vaccine within the next six months?”, “Would you like to encourage your friends to get the HPV vaccine?”, and “What are your main reasons for refusing or discouraging the HPV vaccine?” (Supplementary file 1). In addition, the information in the post-intervention survey was the same as that collected at baseline, except for information on socio-demographic characteristics. The Cronbach’s α value of the questionnaire was 0.816, which achieved good internal reliability.

### Ethical considerations

This study was approved by the Clinical Research Ethics Committee of Peking University Cancer Hospital and the Institutional Review Board of the Chinese Center for Disease Control and Preventive and conformed to the Declaration of Helsinki. Prior to implementation, participants were well informed of the purpose, methods, expected risk, and potential benefits of this study. After that, informed consent was obtained from each participant before the baseline survey.

### Statistical analysis

Categorical data were assessed using case counts and percentages; continuous data, including age and knowledge of HPV vaccine scores, were presented as the mean and standard deviation (SD). Differences between the intervention and control groups and awareness of the HPV vaccine at baseline and post-intervention were analyzed using the *chi*-squared test for categorical data. Student’s *t-*test was used for continuous data. Univariate logistic regression was employed to explore the association of potential factors with the willingness to receive the HPV vaccine. Significant parameters found by univariate analysis (*p* < 0.01) were incorporated and examined using multivariate analysis. Odds ratios (*OR*s) with corresponding 95% (*CI*s) were calculated and reported based on Wald *chi*-square statistics. SPSS statistical software version 20.0 (IBM, Corp, Armonk, New York) was used to analyze the data in this study. Statistical significance was assessed by two-tailed tests with an *α* level of 0.05.

## Results

Nine hundred sixty-seven female college students from two schools were surveyed between February and July 2020. Of these participants, 21 were excluded due to they had received HPV vaccination before the survey. 946 eligible students were enrolled and randomly divided into the control and intervention groups. More than half (56.2%, 532/946) of the enrolled students were in the intervention group and received online health education about HPV and HPV vaccines for seven days. Immediately after the intervention and one month after the intervention, a total of 810 and 820 students completed the post-intervention questionnaire, respectively (Fig. 1).

### Participants’ characteristics

The mean age of the participants was 18.99 ± 0.63 years, and more than half (55.5%) majored in science. The majority of the participants were ethnic Han Chinese (93.0%). A total of 75.5% had lived in urban areas for more than 1 year, and 69.7% of them had monthly living expenses between 1,000 and 2,000 Chinese Yuan (CNY). A total of 35.7% of the students had parents with junior high school or below, 29.5% with senior high school, and 34.9% with a bachelor's degree or above. Furthermore, 79.0% of students had ever received sexual education or knowledge, and 35.1% reported having searched or consulted for HPV vaccines. The vast majority (98.2%) of the students had not had sex, and 84.8% were single. There was no statistically significant difference in general demographic characteristics between the intervention and control groups, except for receiving sexual education or knowledge before the survey (81.8% vs. 74.9%, *p* = 0.010) (Table 1).

**Table 1:** Demographic characteristics of female college students

Variables	All, N (%)	Control group, n (%)	Intervention group, n (%)	*p*
Total participants	967 (100)	414 (43.8)	532 (56.2)	
Research site				
Western	453 (46.8)	177 (42.8)	260 (48.9)	0.061
Central	514 (53.2)	237 (57.2)	272 (51.1)	
Major				
Liberal art	430 (44.5)	174 (42.0)	249 (46.8)	0.143
Science	537 (55.5)	240 (58.0)	283 (53.2)	
Ethnic group				
Han Chinese	899 (93.0)	390 (94.2)	490 (92.1)	0.209
Non-Han Chinese Minority	68 (7.0)	24 (5.8)	42 (7.9)	
Permanent residence place (more than 1 year)				
Urban	730 (75.5)	303 (73.2)	407 (76.5)	0.242
Rural	237 (24.5)	111 (26.8)	125 (23.5)	
Education of parents				
Junior high school or below	345 (35.7)	151 (36.5)	192 (36.1)	0.976
Senior high school	285 (29.5)	121 (29.2)	159 (29.9)	
College and above	337 (34.9)	142 (34.3)	181 (34.0)	
Living expenses per month (CNY^*^)				
< 1,000	201 (20.8)	84 (20.3)	116 (21.8)	0.851
1,000 ~ 2,000	674 (69.7)	293 (70.8)	369 (69.4)	
> 2,000	92 (9.5)	37 (8.9)	47 (8.8)	
Received sexual education				
Yes	764 (79.0)	310 (74.9)	435 (81.8)	0.010
No	203 (21.0)	104 (25.1)	97 (18.2)	
Prior consultation regarding HPV vaccines				
Yes	339 (35.1)	142 (34.3)	177 (33.3)	0.740
No	628 (64.9)	272 (65.7)	355 (66.7)	
Previous sexual experience				
Yes	17 (1.8)	8 (1.9)	9 (1.7)	0.782
No	950 (98.2)	406 (98.1)	523 (98.3)	
Currently relationship				
Yes	147 (15.2)	74 (17.9)	72 (13.5)	0.067
No	820 (84.8)	340 (82.1)	460 (86.5)	

Except for the 21 students who had received the HPV vaccine before the baseline survey, a total of 946 study subjects were randomly assigned to the intervention and control groups; ^*^1 CNY = 0.15 US dollar

### Knowledge and awareness towards HPV and HPV vaccines before and after intervention

Table 2 shows that 63.8%, 66.3%, and 60.8% of the participants had heard of HPV, HPV-related diseases, and HPV vaccines, respectively, before the survey. Nearly 70% of the students knew that persistent HPV infection could lead to cervical cancer. However, knowledge of "HPV infection is almost asymptomatic," and "HPV infection was very common" was relatively poor, with correct rates of 8.3%, and 27.5%, respectively. There was no statistically significant difference in knowledge and awareness of HPV and HPV vaccines between the intervention and control group at baseline, except for correct perception of "persistent HPV infection may lead to cervical cancer" (72.9% vs. 65.7%, *p* = 0.016).

**Table 2:** Knowledge and awareness towards HPV/HPV vaccines between the two groups before and immediately after the intervention

Items	Baseline				Post-intervention			Change percentage point (%)
	All, N (%)	Control, n (%)	Intervention, n (%)	*p*	Control, n (%)	Intervention, n (%)	*p*	
Heard of HPV								
Yes	617 (63.8)	260 (62.8)	337 (63.3)	0.863	335 (89.1)	425 (97.9)	< 0.001	34.6
No	350 (36.2)	154 (37.2)	195 (36.7)		41 (10.9)	9 (2.1)		
Heard of HPV-related diseases (genital warts, cervical cancer, penile cancer)								
Yes	641 (66.3)	275 (66.4)	347 (65.2)	0.700	290 (77.1)	410 (94.5)	< 0.001	29.3
No	326 (33.7)	139 (33.6)	185 (34.8)		86 (22.9)	24 (5.5)		
Heard of HPV vaccine								
Yes	588 (60.8)	237 (57.2)	330 (62.0)	0.136	311 (82.7)	413 (95.2)	< 0.001	33.2
No	379 (39.2)	177 (42.8)	202 (38.0)		65 (17.3)	21 (4.8)		
Persistent HPV infection can cause cervical cancer								
Yes	678 (70.1)	272 (65.7)	388 (72.9)	0.016	270 (71.8)	423 (97.5)	< 0.001	24.6
No/unknown	289 (29.9)	142 (34.3)	144 (27.1)		106 (28.2)	11 (2.5)		
HPV is mainly transmitted through sexual contact intercourse								
Yes	516 (53.4)	205 (49.5)	301 (56.6)	0.031	197 (52.4)	348 (80.2)	< 0.001	23.6
No/unknown	451 (46.6)	209 (50.5)	231 (43.4)		179 (47.6)	86 (19.8)		
Condoms can reduce HPV infection infection								
Yes	345 (35.7)	139 (33.6)	196 (36.8)	0.297	117 (31.1)	210 (48.4)	< 0.001	11.6
No/unknown	622 (64.3)	275 (66.4)	336 (63.2)		259 (68.9)	224 (51.6)		
HPV infection is almost asymptomatic								
Yes	80 (8.3)	38 (9.2)	41 (7.7)	0.417	38 (10.1)	92 (21.2)	< 0.001	13.5
No/unknown	887 (91.7)	376 (90.8)	491 (92.3)		338 (89.9)	342 (78.8)		
HPV infection may result in oral cancer, anal cancer and genital warts								
Yes	547 (56.6)	231 (55.8)	300 (56.4)	0.895	222 (59.0)	393 (90.6)	< 0.001	34.2
No/unknown	420 (43.4)	183 (44.2)	232 (43.6)		154 (41.0)	41 (9.4)		
HPV infection is common								
Yes	266 (27.5)	119 (28.7)	137 (25.8)	0.304	114 (30.3)	342 (78.8)	< 0.001	53.0
No/unknown	701 (72.5)	295 (71.3)	395 (74.2)		262 (69.7)	92 (21.2)		
Ideal time for HPV vaccination is before sex debut								
Yes	483 (49.9)	192 (46.4)	275 (51.7)	0.105	209 (55.6)	417 (96.1)	< 0.001	44.4
No/unknown	484 (50.1)	222 (53.6)	257 (48.3)		167 (44.4)	17 (3.9)		
Knowledge scores of HPV and HPV vaccines	–	2.89 ± 1.93	3.08 ± 1.89	0.381	3.10 ± 1.99	5.13 ± 1.23	< 0.001	/

After the intervention, awareness of HPV, HPV-related diseases, and HPV vaccines in the intervention arm were significantly higher than control arm (*p* < 0.001). In addition, the knowledge score regarding HPV and HPV vaccines was considerably higher in the intervention group than that in the control groups (5.13 ± 1.23 vs. 3.10 ± 1.99, *p* < 0.001). However, the correct rates of "HPV infection is almost asymptomatic" (21.2%) remained low.

### Students' willingness to receive the HPV vaccine and reasons for unwillingness before and after the intervention

**Fig. 2:**
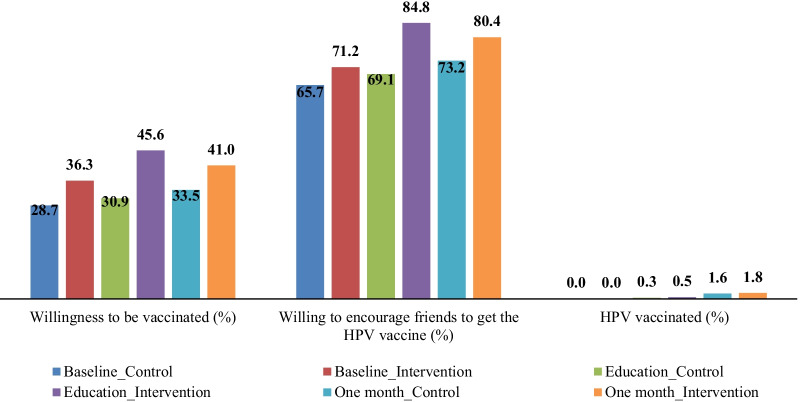
Comparison of intention to vaccinate, encouragement of friends to receive the HPV vaccine, and HPV vaccination between the intervention and control groups at baseline, post-intervention, and one-month follow-up

At baseline, only 2.2% (21/967) of students had received HPV vaccines, and 33.0% (312/946) and 68.8% (651/946) were willing to be vaccinated or willing to encourage their friends to get vaccinated. After the intervention, willingness to be vaccinated and desire to encourage others to take HPV vaccines increased from 36.3% to 45.6% and 71.2% to 84.4%, respectively, in the intervention group. However, the number of vaccinated students remained low, with only 1.6% and 1.8% in the control and intervention groups, respectively (Fig. 2).

The high cost of vaccination (57.7%) and concerns about adverse events (56.0%) were the main reasons for reluctance to be vaccinated at baseline. After the intervention, students in the intervention group had significantly fewer concerns about adverse events (45.5% vs. 60.2%, *p* = 0.001) and the safety and efficacy of the vaccine (26.8% vs. 42.9%, *p* < 0.001) than the control group, but the high price remained the main factor for students to refuse HPV vaccination (Table 3).

**Table 3:** Reasons for reluctance for HPV vaccination between the intervention and control groups before and after the intervention

Reasons for against HPV vaccination	Pre-intervention				Post-intervention		
	All, n (%)	Control group, n (%)	Intervention group, n (%)	*p*	Control group, n (%)	Intervention group, n (%)	*p*
Expense of vaccines	366 (57.7)	168 (56.9)	198 (58.4)	0.711	152 (58.7)	160 (68.1)	0.031
Possible adverse events of HPV vaccine	355 (56.0)	167 (56.6)	188 (55.5)	0.771	156 (60.2)	107 (45.5)	0.001
No sexual activity	243 (38.3)	112 (38.0)	131 (38.6)	0.861	93 (35.9)	95 (40.4)	0.302
Doubts on safety and efficacy of HPV vaccine	237 (37.4)	111 (37.6)	126 (37.2)	0.905	111 (42.9)	63 (26.8)	< 0.001
Fear of needling pain	133 (21.0)	68 (23.1)	65 (19.2)	0.232	59 (22.8)	50 (21.3)	0.687
Vaccination is troublesome	130 (20.5)	51 (17.3)	79 (23.3)	0.061	40 (15.4)	46 (19.6)	0.227

### Factors related to the intention of HPV vaccination

At baseline, five variables were associated with the intention to receive HPV vaccination, as suggested by multivariable logistic regression analysis. Female students from Western China (*AOR*: 1.51, 95% *CI* 1.13–2.02) and students with highly educated parents (*AOR*: 1.91, 95% *CI* 1.34–2.73) were more likely to be vaccinated against HPV. In addition, students who had consulted regarding HPV vaccines (*AOR*: 1.75, 95% *CI* 1.27–2.41) had previous sexual experience (*AOR*: 5.04, 95% *CI* 1.56–16.32) and had heard of HPV vaccines (*AOR*: 1.94, 95% *CI* 1.39–2.71) were more likely to receive the HPV vaccination. Six factors, including residence place, living expenses, sexual education history, current relationship, heard of HPV, and HPV-related disease, were excluded from the multivariable logistic regression model (Table 4).

**Table 4:** Factors associated with willingness to be vaccinated with HPV vaccines among female college students at baseline

Variables	Total, n	Willing to vaccinate, n (%)	*OR* (95% *CI*)	*AOR* (95% *CI*)	*p*
Research site					
Central	509	138 (27.1)	1.00	1.00	
Western	437	174 (39.8)	1.78 (1.35–2.34)	1.51 (1.13–2.02)	0.006
Permanent residence place (> 1 year)					
Rural	236	56 (23.7)	1.00		
Urban	710	256 (36.1)	1.81 (1.29–2.54)		
Education of parents					
Junior high school or below	343	82 (23.9)	1.00	1.00	0.001
Senior high school	280	86 (30.7)	1.41 (0.99–2.01)	1.20 (0.83–1.74)	0.331
College and above	323	144 (44.6)	2.56 (1.84–3.57)	1.91 (1.34–2.73)	< 0.001
Living expenses per month (CNY^*^)					
< 1,000	200	48 (24.0)	1.00		
1,000 ~ 2,000	662	221 (33.4)	1.59 (1.10–2.28)		
> 2,000	84	43 (51.2)	3.32 (1.94–5.68)		
Received sexual education					
No	201	48 (23.9)	1.00		
Yes	745	264 (35.4)	1.75 (1.22–2.50)		
Prior consultation regarding HPV vaccines					
No	627	159 (25.4)	1.00	1.00	
Yes	319	153 (48.0)	2.71 (2.04–3.60)	1.75 (1.27–2.41)	0.001
Previous sexual experience					
No	929	299 (32.2)	1.00	1.00	
Yes	17	13 (76.5)	6.85 (2.21–21.18)	5.04 (1.56–16.32)	0.007
Currently relationship					
No	800	251 (31.4)	1.00		
Yes	146	61 (41.8)	1.57 (1.09–2.25)		
Heard of HPV					
No	349	74 (21.2)	1.00		
Yes	597	238 (39.9)	2.46 (1.82–3.34)		
Heard of HPV-related disease					
No	324	77 (23.8)	1.00		
Yes	622	235 (37.8)	1.95 (1.44–2.64)		
Heard of HPV vaccine					
No	379	76 (20.1)	1.00	1.00	
Yes	567	236 (41.6)	2.84 (2.10–3.84)	1.94 (1.39–2.71)	< 0.001

^*^1 CNY = 0.15 US dollar

OR, odds ratio; AOR, adjusted odds ratio; CIs: confidence intervals

## Discussion

To our knowledge, this current study was the first web-based educational intervention study of HPV vaccination among female college students in the Chinese mainland. Compared with previous studies, this web-based education was independent of time and space and appeared to be as effective as traditional education intervention. Also, it would be easier to promote this educational intervention because of the widespread use of mobile technology [15]. Moreover, this study explored not only the immediate effects after the intervention, but also the long-term effects on both vaccination intention and vaccination behavior after a one-month interval. Last, we adopted an interactive narrative to educate students, rather than just textual or graphic information, to help boost students' willingness to get the HPV vaccine and their vaccination behavior [16].

Consistent with our hypothesis, after seven days of education, female college students in the intervention group showed a significant increase in awareness and knowledge of HPV, HPV-related diseases, and HPV vaccination. This scenario indicated that web-based education could draw the attention of female college students to related issues. Previous studies showed that education was reliable and effective in improving HPV-related knowledge among college students [17, 18] and increasing their willingness to vaccinate against HPV [19]. Notably, although lower than the intervention group, we also observed an increased awareness and knowledge regarding HPV vaccines among the control group, which might be attributed to the following reasons. First, filling out the questionnaire may help to popularize the concept of the HPV vaccine, leading to self-learning. Second, the HPV vaccine has been available in the Chinese mainland for more than 5 years, and a large amount of information was readily available to assist students in their self-study [5, 20, 21].

Despite improving HPV vaccine-related knowledge, our study's cumulative HPV vaccine appointment/vaccination rate was meager one month after the 7-day intervention. A study conducted among African American girls found similar results in that changes in knowledge did not lead to behavioral action [22]. This suggests that when implementing health education for the HPV vaccine, we should not only talk about the knowledge but also provide information about vaccination behaviors and techniques, such as where to get vaccinated and how to get the HPV vaccine. Moreover, the present study was conducted during the COVID-19 pandemic, and it was difficult for the participants to implement vaccination. Thus, many students did not receive the HPV vaccine despite their willingness to do so. Additionally, there is a need to consider the supply and accessibility issues of HPV vaccines since HPV vaccines are not yet available in many rural areas in the Chinese mainland [23], and some regions were forbidden from distributing the HPV vaccine during the COVID-19 pandemic. Finally, this study followed up for only one month; future studies with longer follow-up are needed to observe changes in the behavior of the study subjects.

It was worth noting that the intervention group’s motivation to get vaccinated and encourage friends increased significantly immediately after the intervention and decreased after one month. A PPT-oriented educational study conducted among middle school girls showed that the perceived level in the intervention group was obviously lower than that immediately after the intervention due to the lack of a boosting health education during the 1-year interval [24]. These findings suggested that a single, short-term health education does have a positive effect on female college students’ attitudes toward HPV vaccination; however, this effect gradually diminished over time. Thus, a long-term health education strategy integrated into the school-based curriculum might significantly improve HPV vaccination uptake. Currently, the prevention of HIV/AIDS has been integrated into the sexual health education curriculum for school students in the mainland of China [25]. As HPV is also a sexually transmitted infection, it is recommended to include HPV-associated health education in students' sexual health education curriculum.

In the current study, only 2.2% of female college students had received HPV vaccination before the survey, which was much lower than female college students in Beijing (9.5%) [26], Hong Kong (13.3%) [27], and the developed eastern regions of China (13.7%) [10]. Meanwhile, 33.1% of the responders reported being willing to vaccinate themselves, much lower than the national average (53.5%) [10]. In this study, students with highly educated parents seemed to be more willing to receive the HPV vaccination. Parents are the key decision-makers for their children's HPV vaccination has been widely reported [28, 29], highlighting the significance of training for parents along with training for students. Moreover, students who reported having sexual experience were more likely to initiate HPV vaccination, possibly because sexual experiences led to more knowledge and awareness of sexually transmitted diseases and their associated knowledge [30]. In this study, only 1.7% of female college students had sexual experience before the survey, significantly lower than the reported 17.8% in previous studies [10]. This may be because the current study participants were female freshmen, who might have more conservative sexual attitudes than upper-year students. Notably, in our study, 21.0% of students reported not receiving any sexual education before the survey, indicating that sexual education still needs to be strengthened in mainland China.

HPV vaccination is relatively expensive for most college students in the presented study, which leads to various affordability issues with the increasing demand for HPV vaccination. Currently, the available imported bivalent, quadrivalent, and 9-valent HPV vaccines in the Chinese mainland cost about 600 CNY, 800 CNY, and 1,300 CNY each dose, respectively, and are too expensive to afford for most college students [31]. Besides, the domestic bivalent HPV vaccine has been approved for marketing [32]. It costs 329 CNY per dose, which is much lower than the imported HPV vaccines, making it expected to increase the vaccination rate of students. In the meantime, authorities should also consider including HPV vaccines in the Chinese national immunization program to solve low vaccination rates due to the high price of HPV vaccines. In addition, concerns about the adverse events, safety, and efficacy of the vaccine are also reasons students are reluctant to get vaccinated. Many studies have proven the safety and efficacy of the HPV vaccine [32–34], and its adverse events have been clarified [35]. Therefore, further education should enhance the safety, efficacy, and adverse events of HPV vaccines for female college students to eliminate their doubts and promote their vaccination against HPV.

Nevertheless, the study has several limitations. First, this study enrolled only two universities from two cities, which cannot accommodate representative female college students in Western and Central China. Future studies with broader coverage and larger sample sizes are needed to provide evidence for HPV vaccination promotion. Second, since all questionnaires were self-reported, some results should be interpreted with caution, especially questions about sexual activities. Third, all students in the intervention group were included in the post-intervention analysis but did not assess the completion and quality of student learning, thus potentially underestimating the effect of the intervention. Fourth, a significantly higher proportion of students in the intervention group had received sexual health education than in the control group (81.8% vs. 74.9%, *p* = 0.010) before health education, which may underestimate the effect of the intervention. Last, the study was conducted during the COVID-19 outbreak, and there were barriers to HPV vaccination, resulting in no significant behavioral changes observed in this study.

## Conclusion

In summary, female college students’ HPV vaccination uptake is deficient, and they have minimal detailed knowledge about HPV and its vaccines. Web-based health education on HPV vaccines is an easy, feasible, and effective way to improve the awareness and acceptance of HPV vaccination among female college students but does not change vaccination behavior. Further studies are needed to explore suitable HPV vaccine intervention strategies for female college students and improve HPV vaccination rates in the Chinese mainland.

## Supplementary Information

**Additional file 1.** The questionnaire used in this study.

## Data Availability

The dataset supporting the conclusions of this article is available from the corresponding author on reasonable request.

## Abbreviations

AOR: Adjusted odds ratio
CIs: Confidence Intervals
CNY: Chinese Yuan
HPV: Human papillomavirus
P: Probability
OR: Odds ratio
SD: Standard deviation
WHO: World Health Organization
