# Supplementary material for: Impact of web-based health education on HPV vaccination uptake among college girl students in Western and Northern China: a follow-up study
Source: BMC Womens Health. 2022 Feb 23;22:46. doi: 10.1186/s12905-022-01625-0 (PMC8864209; doi:10.1186/s12905-022-01625-0)
Supplement: Supplementary file 1 — Additional file 1. The questionnaire used in this study. [file 12905_2022_1625_MOESM1_ESM.pdf]

Intervention Study on HPV Vaccination among Female College Students in China

Ⅰ General information

1. Have you been vaccinated against HPV? □Yes □No（If no, go to question 4）

2. Date of vaccination：______/____ month/year

3. Place of vaccination： □Chinese mainland □Other places

4. Date of birth：______/____month/year

5. Ethnic： □Han Chinese □Other_______________

6. Residence (residence for more than 1 year) □Urban area □Rural area

7. Your parents' residence (residence for more than 1 year) □Urban area □Rural area

8. Marital status of parents □Married □Divorce/Separation □Cohabit □Widowed

9. Educational level of parents: □Junior high school or below □Senior high school □Undergraduate or Higher

10. Your living expenses (RMB/month) □ Below 1000 Yuan □ 1000~2000 Yuan □2001~3000yuan □ More than 3000

11. Do you have a close family member, friend or other close person who has cancer? □ Yes □ No

12. Have you heard of HPV? □ Yes □ No

13. Have you heard of HPV-related diseases, such as genital warts, cervical cancer, penile cancer, anal cancer? □ Yes □ No

14. Have you heard of the HPV vaccine? □ Yes □ No

Ⅱ Health and sexual behavior

16. Do you agree or disagree with the statement that vaccines can effectively prevent diseases? □ Yes □ No

17. Have you received any type 2 vaccines (such as hepatitis B vaccine, influenza vaccine)? □ Yes □ No

18. Have you received any sex education or knowledge? □ Yes □ No

19. Have you actively searched for or consulted for HPV vaccines? □ Yes □ No

20. Have you ever had sex? □ Yes □ No （If no, go to question 22）

21. Your first sexual encounter occurred in：_____year
22. Are you single? □ Yes □ No
23. Can you accept premarital sex? □ Yes □ No

Ⅲ **HPV-related knowledge** 24. Please judge whether the following statements are true or false

	True	False	Don’t know
Persistent HPV infection can cause cervical cancer.			
HPV is mainly transmitted through sexual contact.			
Condoms protect against HPV infection.			
HPV infection is usually asymptomatic.			
HPV infection can cause oral cancer, genital warts, and anal cancer.			
HPV infections are usually transient and mostly self-healing.			
HPV infection is very common.			
The best time to get vaccinated is before first debut of sex.			

Ⅳ **HPV vaccination intention**

28. Do you want to get the HPV vaccine within the next 6 months? □ Yes □ No

29. Would you like to encourage your friends to get the HPV vaccine? □ Yes □ No

30. What are the main reasons why you do not want or encourage HPV vaccination?

□Doubt about the safety and effectiveness of the vaccine □Concerned about the side effects of the vaccine

□Fear of needling pain □HPV vaccination is expensive □Three regular vaccinations is too cumbersome

□Not having sex (virgin) □Other, Please specify：_______________
